# Survival outcomes of hybrid total hip replacement following failed proximal femoral nail antirotation: a retrospective study with a median 10-year follow-up

**DOI:** 10.3389/fsurg.2025.1562738

**Published:** 2025-05-13

**Authors:** Yannan Chen, Zhifen Lai, Weiguang Yu, Xianshang Zeng, Mingdong Zhao, Guangquan Zhu

**Affiliations:** ^1^ Department of Anesthesiology, Wuhan Fourth Hospital, Wuhan, China; ^2^ Department of Radiology, The First Affiliated Hospital, Sun Yat-sen University, Guangzhou, China; ^3^ Department of Orthopedics, The First Affiliated Hospital, Sun Yat-sen University, Guangzhou, China; ^4^ Department of Orthopedics, Jinshan Hospital, Fudan University, Shanghai, China; ^5^ Department of Orthopedics, Beijing Shijingshan Hospital of Traditional Medicine, Beijing, China

**Keywords:** fracture, total hip replacement, revision, conversion, outcome

## Abstract

**Background:**

This retrospective study evaluates the efficacy of hybrid total hip replacement (THR) in patients aged 50 to 70 years who have experienced failures following proximal femoral nail antirotation (PFNA) procedures. By addressing a significant gap in the current medical literature—characterized by inadequate data and inconsistencies regarding the effectiveness of hybrid THRs in revision settings—this research aims to provide valuable insights into the long-term viability and clinical outcomes of hybrid THR for this demographic.

**Methods:**

In this retrospective observational study, we investigated 185 individuals aged 50 to 70 years who underwent hybrid THRs following PFNA procedures across two specialized Joint Surgery Centers. The primary objective of this study was to evaluate implant longevity, which was assessed using the Kaplan–Meier method, with a particular focus on revision surgeries. Additionally, we aimed to analyze secondary outcomes, including patient-reported experiences quantified by the EuroQol Visual Analogue Scale (EQ-VAS) and the Likert pain scale. Furthermore, this study sought to quantify the rates of major orthopedic complications within this patient cohort.

**Results:**

A total of 124 individuals (124 THRs) were assessed, resulting in a median follow-up duration of 10 years (range: 3–15 years). The 10-year survivorship, defined as the rate of survival without revision for any reason, was found to be 87.1% (78.5%–90.1%). Stratified survival analysis by age groups (50–60 years and 60–70 years) revealed that the 50–60-year group had significantly higher survival rates compared to the 60–70-year group (*p* = 0.00026). Postoperative pain scores averaged 3.0 (95% CI, 2.9–3.1), indicating a significant reduction in pain. Furthermore, patient satisfaction was high, with an average satisfaction score of 3.7 (95% CI, 3.6–3.8). The mean EQ-VAS score was 77.4 (95% CI, 76.4–78.3), reflecting favorable post-surgical health perceptions. Among the 124 patients, 13 experienced a total of 19 implant-related complications, leading to an incidence rate of 10.4% for major orthopedic complications.

**Conclusion:**

Hybrid THR shows durable efficacy in patients aged 50–70 with failed PFNA, achieving high revision-free survival and improved postoperative outcomes. Younger patients (50–60 years) had superior survival, while Staphylococcus/Enterococcus infections worsened prognosis. Non-infected individuals aged 50–60 achieved optimal 10-year survival. Complications like stem loosening were reduced, but cement degradation and infection risks remain challenges. Future efforts should target age-specific protocols and infection mitigation.

## Introduction

The increasing challenge of managing failed proximal femoral nail antirotation (PFNA) procedures in China's aging population necessitates a comprehensive review of revision strategies in orthopedic surgery, particularly for patients who are often burdened with osteoporosis and comorbidities (1–4). The prevalence of PFNA failures, which significantly compromise quality of life, has resulted in a notable rise in revision surgeries, sparking discussions about the most effective approaches, such as hybrid, cemented, or uncemented total hip replacements (THRs) (5–7). Recent trends indicate a growing preference for hybrid THR, which is recognized for its superior functional outcomes and reduced orthopedic complications compared to other THR modalities (8, 9). This approach provides better initial stability following revision, facilitating earlier weight-bearing and yielding promising clinical results. Nonetheless, concerns persist regarding the adverse effects associated with cemented components, particularly cement-induced osteolysis, which can contribute to prosthesis failure (10, 11). Therefore, the choice between hybrid, cemented, or uncemented THRs is critical for patients, significantly impacting treatment outcomes and necessitating meticulous consideration of each patient's individual medical condition and lifestyle (12, 13).

In China, the preference for hybrid THRs among patients aged 50 to 70 years is on the rise, largely due to advancements in cement fixation technologies (5). However, uncertainties persist regarding the long-term viability of hybrid THRs, particularly given the lack of extensive follow-up data extending beyond five years for Chinese patients who have undergone hybrid THR following failed PFNA procedures (5, 14). To address this critical knowledge gap, our retrospective study seeks to evaluate the long-term outcomes of hybrid THR in patients aged 50 to 70 years with prior PFNA failures, thereby providing essential insights into the effectiveness of this technique within this specific age group. This study highlights the necessity for a comprehensive, patient-centered approach when determining the most suitable type of THR for these patients.

## Materials and methods

### Study population

From March 2005 to December 2023, a retrospective cohort analysis was conducted involving patients aged 50 to 70 years who underwent hybrid THR following failed PFNA at two specialized joint trauma centers. These centers reported a median annual volume of 20 THR revision procedures, with a range of 11 to 32. Detailed information regarding the devices utilized in both PFNA and hybrid THR procedures can be found in Table 1. The reasons and types for PFNA revisions were meticulously extracted from electronic medical records.

**Table 1:** Product specifications for PFNA and hybrid THR.

Parameter	Stem^a^	Cup^a^	PFNA^b^
Hybrid THR (*n* = 124)	Cemented stem with ceramic femoral head	Uncemented monoblock trabecular metal cup^b^	Synthes, Solothurn, Switzerland

^a^ Zimmer, Warsaw, Indiana.

^b^ Highly porous tantalum with a polyethylene liner.

PFNA, proximal femoral nail antirotation; THR, total hip replacement.

Patient comorbidities were evaluated using the Charlson Comorbidity Index (CCI). The inclusion criteria specifically targeted patients aged 50 to 70 years with a history of initial PFNA fixation that necessitated subsequent hybrid THR revision. Strict exclusion criteria were applied to ensure robust study integrity, which involved excluding cases lacking essential demographic information (such as diagnosis, fixation type, and implant details), as well as those with hip deformities, loss of independent mobility, prior contralateral intertrochanteric fractures, lower extremity neurological disorders, advanced tumors, active infections (including sepsis), mental health disorders (such as schizophrenia and intellectual disabilities), long-term dialysis, or pharmacological treatments for conditions like renal failure and immunosuppression. Additionally, non-compliance with the prescribed follow-up regimen was a key exclusion criterion.

### Surgical procedures

In the transition from PFNA to hybrid THR, our methodical approach began with the systematic removal of the PFNA device. We meticulously measured the distance from the lesser trochanter to the distal tip of the main nail, ensuring that the selected stem length at least matched this measurement. This precaution was crucial for mitigating the risk of periprosthetic fractures resulting from stress concentration. The conversion procedures were consistently performed using a lateral approach. Following the truncation of the femoral neck at the femoral head-neck junction and the base of the femoral neck, we carefully excised the glenoid labrum and polished the acetabulum until hemostasis was achieved. The uncemented cup insertion was carried out in accordance with manufacturer guidelines, maintaining a valgus angle of 40° to 45° and an anteversion angle of 15° for precise placement of the acetabular cup, followed by secure affixation of the liner.

For the insertion of cemented stems, we employed third-generation cementing techniques (15), effectively addressing complications such as subperiosteal osteolysis, femoral calcar resorption, proximal femoral bone defects, and disruption of the greater trochanter's integrity. Key procedural steps included the selection of a long-stem prosthesis, the utilization of wedge-shaped bone masses from the femoral neck for reconstruction of the greater trochanter, and the generation of small bone fragments from the femoral head and neck for grafting purposes. Cortical defects were managed using steel cables and metal mesh. The proximal femoral medullary cavity was thoroughly cleansed, with precise positioning of a long guide needle followed by the implantation of bone fragments into the medullary cavity and the residual lateral screw holes after PFNA removal.

Postoperatively, our focus shifted to pain management, physiotherapy, and monitoring for complications, emphasizing early supervised mobilization. Patients adhered to a standardized rehabilitation protocol (16), initiating range-of-motion exercises immediately after surgery and progressing to full weight-bearing status within three months. To mitigate the risk of infection, a regimen of first-generation cephalosporins was administered preoperatively and continued for 24 h post-conversion, supplemented by routine antithrombotic prophylaxis to prevent thromboembolic events.

### Outcomes and variables

The primary endpoint of this study, implant survivorship, was assessed using the Kaplan–Meier method, defining revision for any reason as the endpoint of interest. The criteria for revision included the exchange or removal of any implant component due to symptomatic issues, irrespective of any adjustments made (17). The reasons for converting to hybrid THR included nail breakage, implant cutout, periprosthetic fracture, and nonunion. Secondary endpoints comprised patient-reported outcome measures (PROMs) and critical orthopedic complications, notably aseptic loosening, dislocation, and periprosthetic fracture. PROMs were evaluated utilizing the EuroQol Visual Analogue Scale (EQ-VAS) (18) and the Likert pain scale (19), which assessed health status and pain both preoperatively and postoperatively. EQ-VAS scores ranged from 0 to 100, while Likert scores at the final follow-up measured patient satisfaction and the overall impact of the surgery. Stem loosening was identified by observing progressive radiolucent lines on sequential x-rays, with acetabular component loosening indicated by a continuous line exceeding 2 mm on both anteroposterior and lateral views (20). Criteria for cup loosening were based on the presence of complete radiolucent lines, changes in inclination exceeding 5°, or migration greater than 5 mm (21). Recurrent dislocation was defined as more than three instances within six months, and implant infection adhered to the 2018 periprosthetic joint infection standards (22). Imaging was centrally reviewed at each follow-up, with secondary endpoints confirmed by co-authors WY and XZ at the conclusion of the study. Orthopedic complications were documented throughout the follow-up period, which extended from revision of PFNA to THR, death, or study completion. Patient evaluations were conducted bi-monthly, either in person or via phone.

### Statistical analysis

Data analysis consisted of median and frequency reporting for continuous and categorical variables, respectively. The timing of revisions post-conversion was analyzed using Kaplan–Meier and log-rank methods. Descriptive statistics provided an overview of complications and follow-up durations, with the latter analyzed using reverse Kaplan–Meier methodologies. We employed Cox proportional hazards models to calculate median survival, treating revision THR as a time-dependent variable and accounting for baseline covariates, with death considered a competing event as per established guidelines. A statistical significance threshold was set at *p* < 0.05, with analyses performed using SAS 9.4 and R software (version, 4.4.2).

## Results

### Clinical characteristics of patients

**Figure 1:**
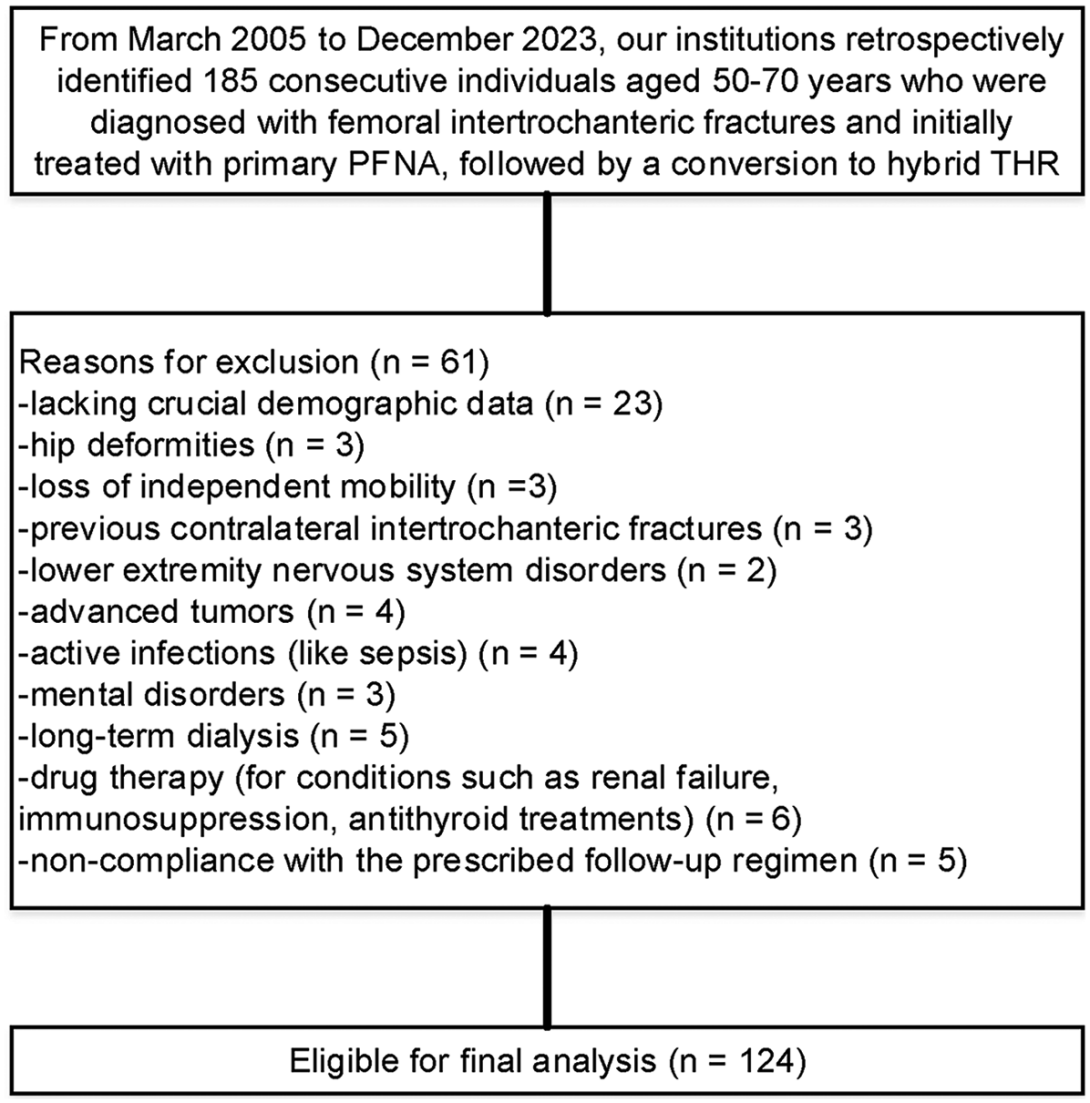
Flow diagram delineating the methodology for identifying study subjects, assessing the survival outcomes of hybrid THRs post prior PFNA failure.

**Figure 2:**
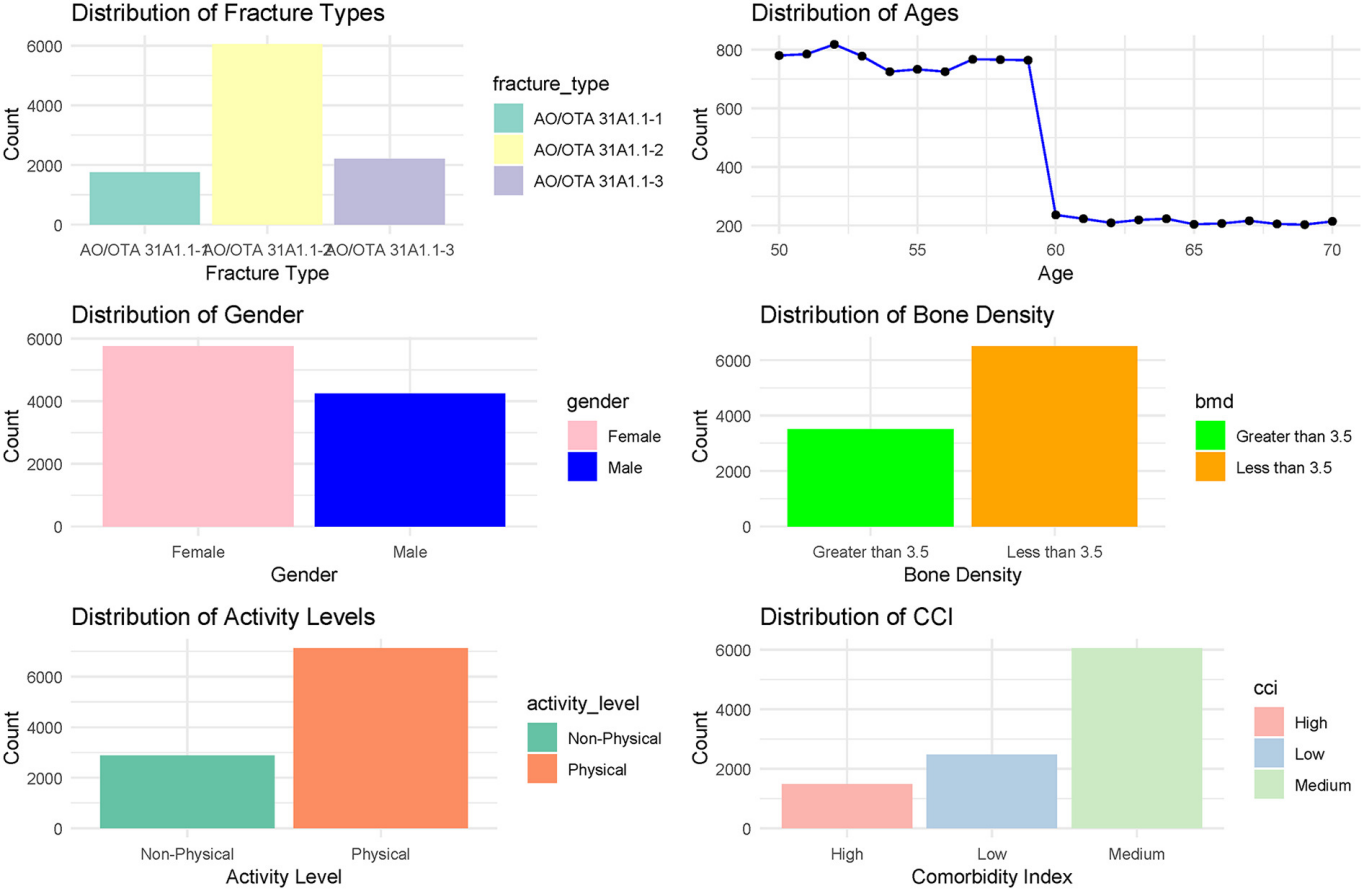
The main characteristics of revision patients in this study.

**Figure 3:**
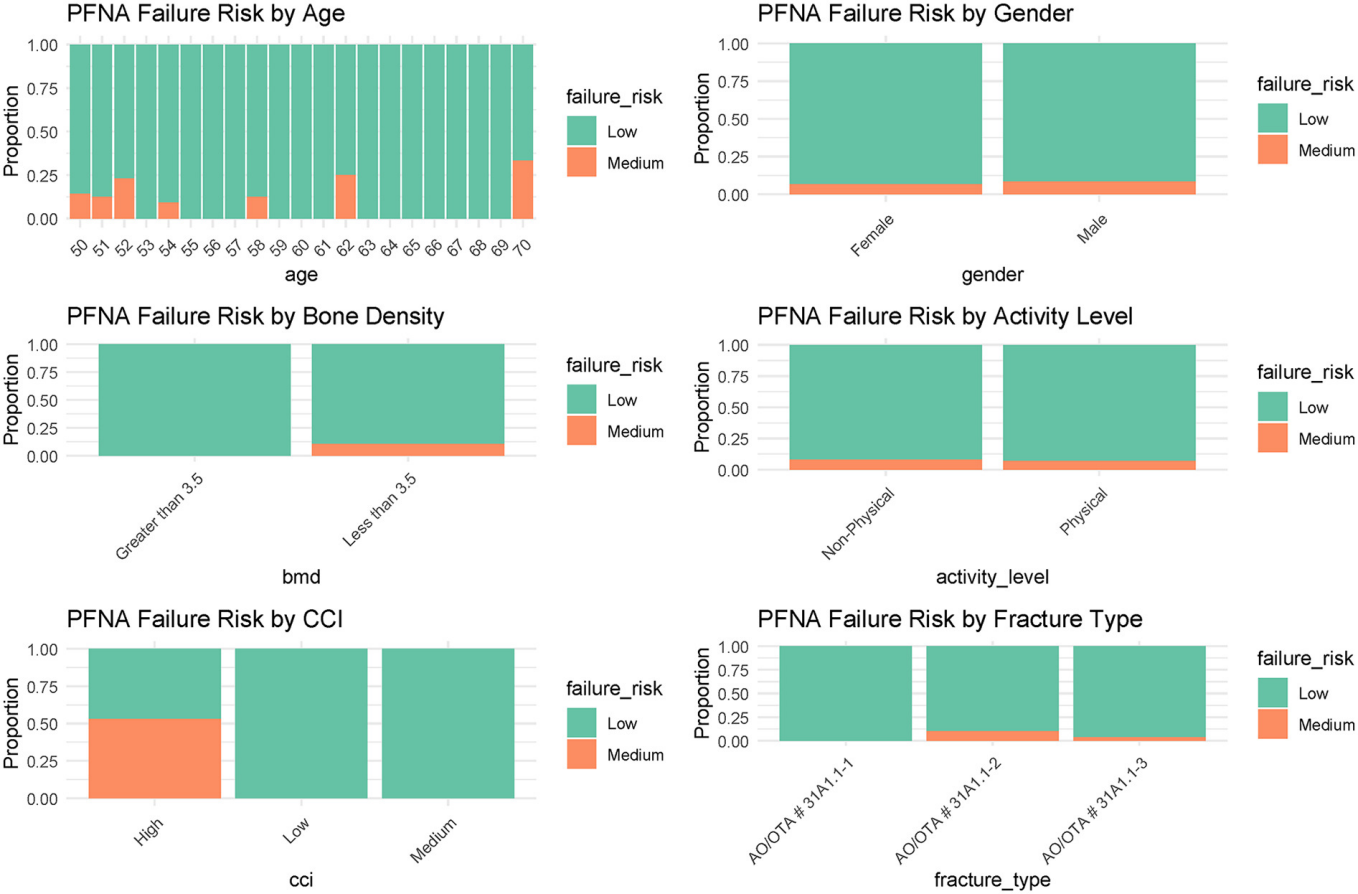
The proportion of revisions corresponding to the main baseline characteristics.

Initially, 185 patients aged 50 to 70 years were assessed for inclusion in the study, of whom 61 were excluded based on our predefined criteria. The remaining 124 patients, all of whom underwent hybrid THR, constituted the final study cohort (Figure 1). Detailed baseline characteristics are presented in Table 2, indicating that a majority of the participants (75.8%) were aged 50 to 60 years. The cohort exhibited a slightly higher female representation (58.0%) compared to males (42.0%). The body mass index (BMI) had a median of 22.4 (range: 19.5 to 32.7), while the bone mineral density (BMD) of the proximal femur showed a median value of 3.5 (range: 3.0 to 4.8). Regarding injury laterality, the distribution was relatively balanced, with left-side injuries accounting for 51.6% and right-side injuries 48.4%. The most common indications for the initial surgeries were categorized under AO/OTA codes, with 59.7% classified as AO/OTA 31A1.2. The predominant mechanisms of injury were falls (62.9%), followed by tamp injuries (26.6%) and traffic accidents (10.5%). The median time to hybrid THR conversion was within 6 months for 62.9% of cases. Furthermore, the CCI at the time of revision indicated a prevailing medium grade (58.9%), reflecting a significant burden of comorbidities in this population. The primary indications for conversion to hybrid THR were nail breakage (36.3%), cutout (29.0%), periprosthetic fracture (26.6%), and nonunion (8.1%). Most patients were classified as ASA physical status 2 (59.7%), indicating the presence of mild systemic disease. The main characteristics of these revision patients are shown in Figure 2, along with the corresponding proportions of revision risks depicted in Figure 3.

**Table 2:** Baseline characteristics of patients who underwent hybrid THR following failed PFNA.

Variable	Hybrid THR (*n* = 124)
Age (years), no. %	
50 ≤, <60	94 (75.8)
60 ≤, ≤70	30 (24.2)
Sex, no. %	
Female	72 (58.0)
Male	52 (42.0)
BMI (kg/m^2^)	
Median (range)	22.4 (19.5–32.7)
BMD (proximal femur) (g/cm^3^)	
Median (range)	3.5 (3.0–4.8)
Side, no.%	
Left	64 (51.6)
Right	60 (48.4)
Reason of primary surgery, no.%	
AO/OTA 31A1.1	22 (17.7)
AO/OTA 31A1.2	74 (59.7)
AO/OTA 31A1.3	28 (22.6)
Mechanism of injury, no.%	
Traffic	13 (10.5)
Falling	78 (62.9)
Tamp	33 (26.6)
Time to hybrid THR conversion (months), no.%	
<6	78 (62.9)
≥6	46 (37.1)
CCI at revision, no. %	
Low	32 (25.8)
Medium	73 (58.9)
High	19 (15.3)
Indications for conversion to hybrid THR, no. %	
Nail breakage	45 (36.3)
Cutout	36 (29.0)
Periprosthetic fracture	33 (26.6)
Nonunion	10 (8.1)
ASA physical status, no.%	
1	26 (20.9)
2	74 (59.7)
3	24 (19.4)

THR, total hip replacement; BMI, body mass index; BMD, bone mineral density; CCI, Charlson comorbidity index; ASA, American society of anesthesiologists.

### Primary outcome

**Figure 4:**
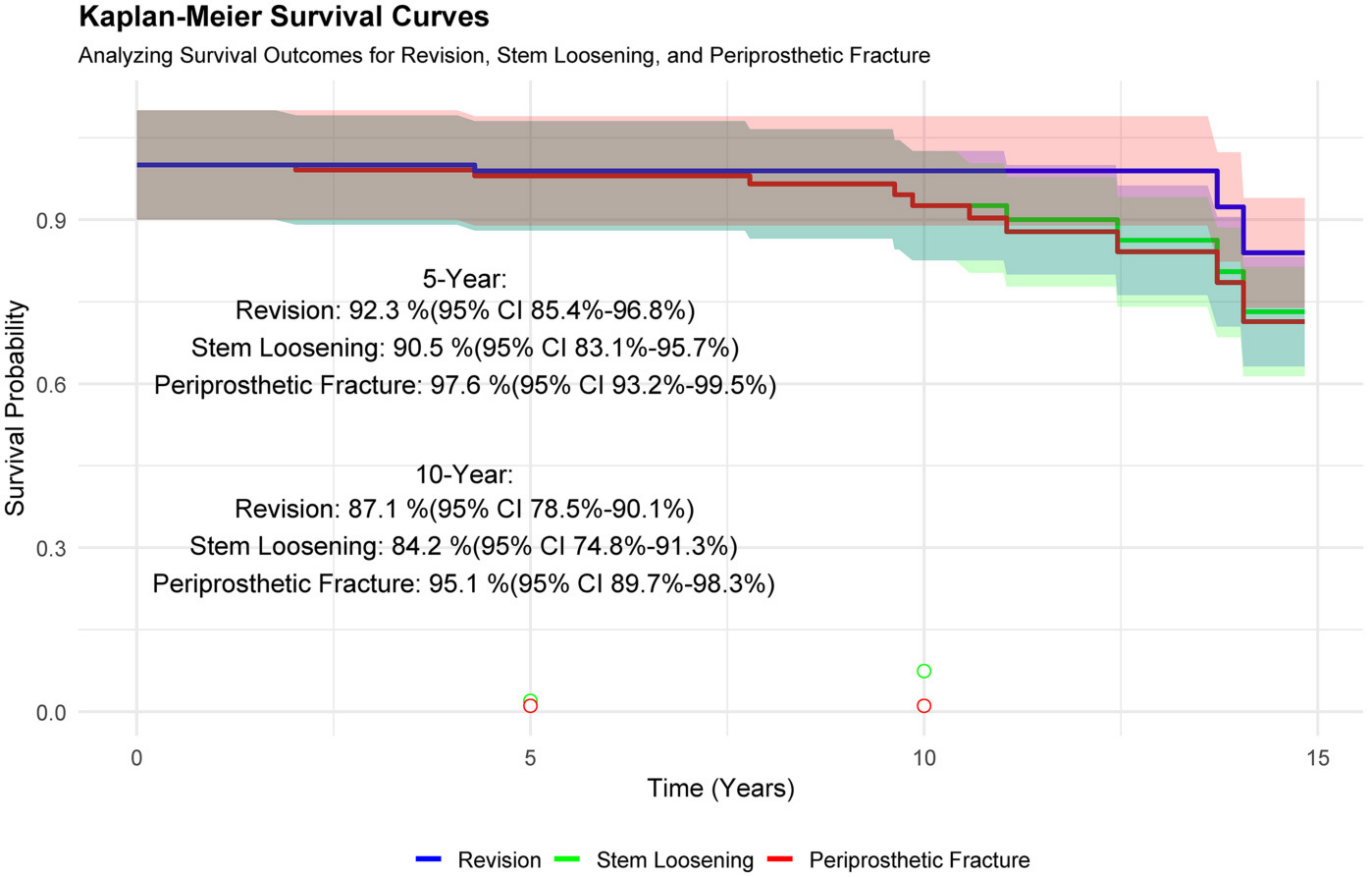
Survival analysis was conducted using kaplan–meier curves, with revision THR, stem loosening, and periprosthetic fracture serving as endpoints to evaluate the longevity of the prosthesis under different conditions.

The median follow-up period for this study exceeded 10 years, with a range from 3 to 15 years. The Kaplan–Meier survival analysis presented in Figure 4 evaluates long-term outcomes for three adverse events following hybrid THR: revision, stem loosening, and periprosthetic fracture. At 5 years, survival probabilities (with 95% CI) were 92.3% (85.4%–96.8%) for revision, 90.5% (83.1%–95.7%) for stem loosening, and 97.6% (93.2%–99.5%) for periprosthetic fracture. By 10 years, survival rates declined to 87.1% (78.5%–90.1%) for revision, 84.2% (74.8%–91.3%) for stem loosening, and 95.1% (89.7%–98.3%) for periprosthetic fracture. These results indicate a gradual decrease in survival probabilities over time, with revision and stem loosening showing steeper declines compared to periprosthetic fracture, which maintained the highest survival rate at both time points. The overlapping CIs suggest variability in long-term outcomes, particularly for stem loosening and revision. Among the 124 patients who received hybrid THRs, 115 (92.7%) did not require revision surgery. The predominant causes for the few revisions included stem loosening, accounting for 66.7% of cases, and periprosthetic fractures, which were responsible for the remaining 33.3%. Notably, while dislocations occurred relatively frequently, they did not necessitate revisions, thus not impacting the overall prosthesis survival rate within our cohort.

**Figure 5:**
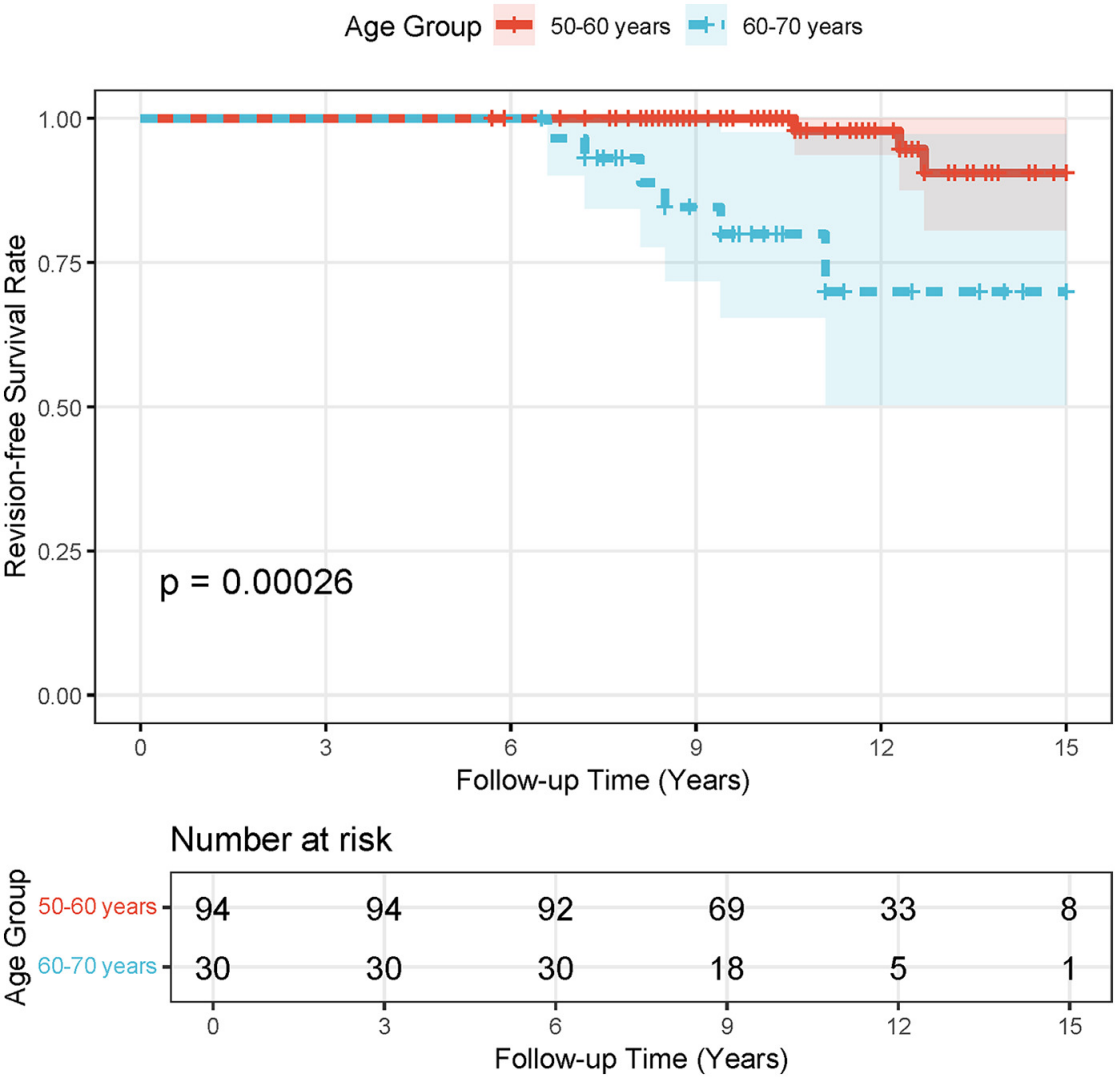
Stratified survival analysis by age groups (50–60 years and 60–70 years) was performed using kaplan–meier curves, with revision THR as the endpoint to assess the longevity of the prosthesis.

The Kaplan–Meier survival analysis in Figure 5 compares postoperative survival outcomes between two age groups (50–60 years and 60–70 years) over a 15-year follow-up period. Survival probabilities declined gradually for both cohorts, with the 50–60-year group demonstrating consistently higher survival rates compared to the 60–70-year group (log-rank test, *p* = 0.00026), indicating statistically significant differences in outcomes. At baseline, the number at risk was 94 for the younger group and 30 for the older group. By year 15, the younger cohort retained 8 patients at risk compared to 1 in the older group. These results suggest that age at surgery significantly influences long-term survival, with younger patients (50–60 years) exhibiting more favorable outcomes over time. The widening gap in survival probabilities highlights the clinical relevance of age stratification in postoperative risk assessment.

**Figure 6:**
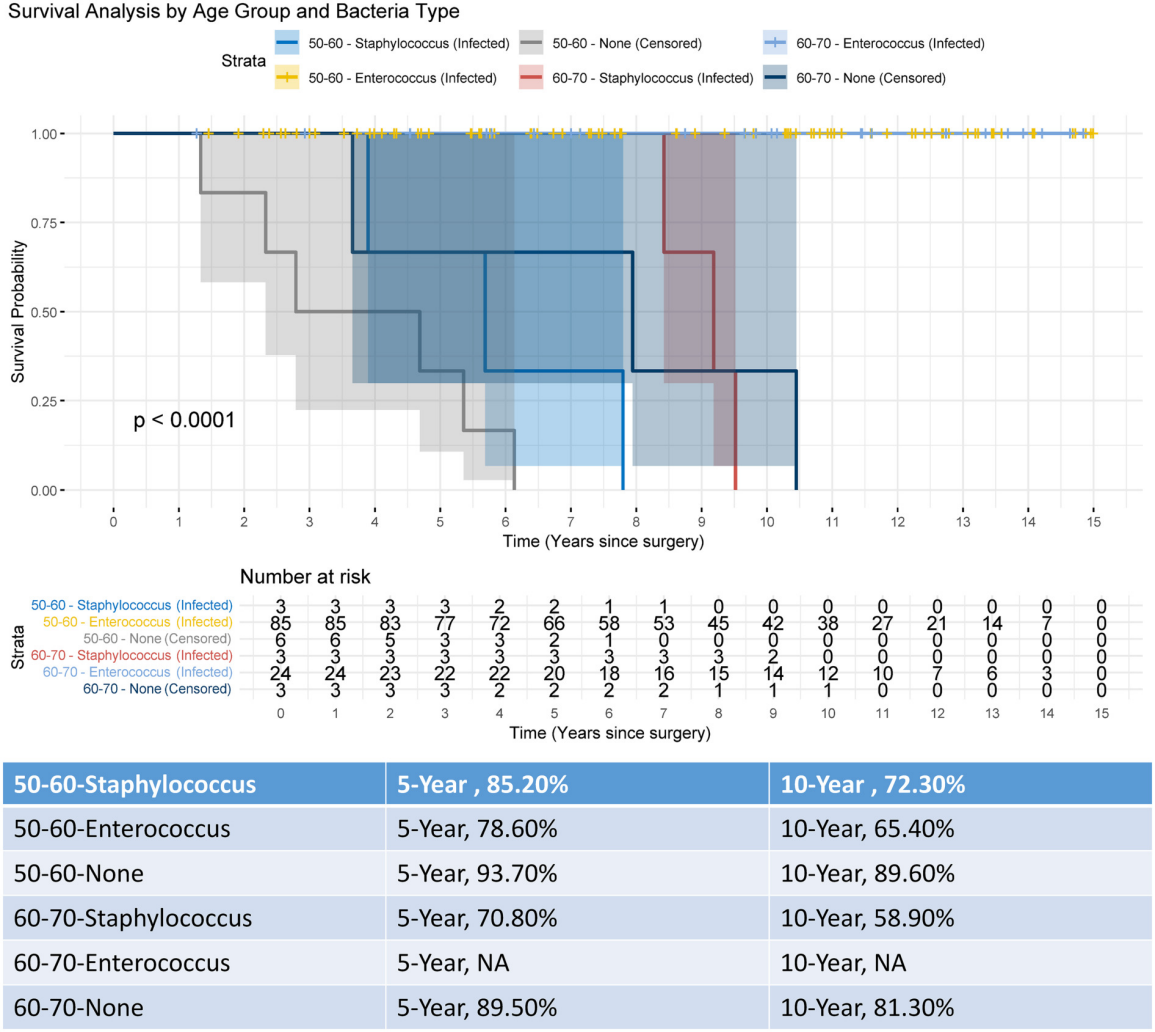
Kaplan–meier survival analysis stratified by age group and bacterial infection status over a 15-year follow-up.

Figure 6 presents a comprehensive survival analysis stratified by age groups and bacterial infection status following surgical intervention. The Kaplan–Meier survival curves demonstrate distinct trajectories across strata, with a statistically significant divergence in survival probabilities over a 15-year follow-up period (log-rank *p* < 0.0001). The 50–60-year-old cohort infected with Staphylococcus exhibited a progressive decline in survival probability from 1.00 to 0.00, whereas the non-infected (censored) subgroup within the same age range displayed superior survival outcomes. Notably, the 60–70-year-old cohort infected with Enterococcus showed the steepest survival decline, likely attributable to limited sample size and heightened vulnerability in this subgroup. Survival rate comparisons at 5- and 10-year intervals further emphasize clinical implications: non-infected individuals aged 50–60 achieved the highest survival rates (5-year: 93.7%; 10-year: 89.6%), contrasting sharply with Staphylococcus-infected peers (5-year: 85.2%; 10-year: 72.3%). The absence of survival data (*N*A) for the 60–70-year-old Enterococcus-infected group underscores potential limitations in long-term follow-up or statistical power due to minimal at-risk participants (*n* = 3 at baseline). These findings collectively underscore the prognostic significance of age, pathogen type, and infection status in post-surgical survival outcomes.

### Secondary outcomes

Patient-reported outcomes, meticulously detailed in Table 3, include various metrics assessed at the final follow-up to evaluate the efficacy of the procedure. The mean pain score, a critical indicator of postoperative pain relief, was quantified at 3.0 (95% CI, 2.9–3.1), reflecting a significant reduction in pain levels. Additionally, patient satisfaction, another vital outcome, was measured with a mean satisfaction score of 3.7 (95% CI, 3.6–3.8), indicating a high degree of contentment with the results of the intervention. Furthermore, the mean EQ-VAS score, representing patients' overall health status, was found to be 77.4 (95% CI, 76.4–78.3), denoting a favorable perception of health following surgery. Table 4 outlines significant implant-related complications, reporting that at the final follow-up, 9 individuals (7.2%) underwent conversion from hybrid THR to revision surgery. Stem revision was the most prevalent (4.8%), followed by acetabular revision (1.6%) and combined revisions (0.8%). The primary complications included stem loosening (8.0%), dislocation (4.8%), and periprosthetic fracture (2.4%). Out of 124 patients, 13 experienced 19 implant-related complications, leading to an overall incidence of key orthopedic complications of 10.4% (13/124).

**Table 3:** Measurement of pain distribution and levels of satisfaction, utilizing the EQ-VAS on a Likert scale, accompanied by a VAS that spans from 0 to 100.

Measurement	Hybrid THR (*n* = 124)
Pain, preoperatively, n%	
1 none	12 (9.6)
2 very mild	28 (22.5)
3 mild	35 (27.3)
4 moderate	42 (33.8)
5 severe	7 (5.6)
Mean score (CI)	3.0 (2.9–3.1)
Pain, final follow-up, *n*%	
1 none	42 (33.8)
2 very mild	30 (24.1)
3 mild	27 (21.7)
4 moderate	20 (16.1)
5 severe	5 (4.0)
Mean score (CI)	2.3 (2.2–2.5)
Satisfaction, final follow-up, *n*%	
1 very dissatisfied	12 (9.6)
2 dissatisfied	12 (9.6)
3 neither nor	22 (17.7)
4 satisfied	34 (27.4)
5 very satisfied	44 (35.4)
Mean score (CI)	3.7 (3.6–3.8)
EQ-VAS, preoperatively	
Mean score (CI)	52.6 (50.5–53.6)
EQ-VAS, final follow-up	
Mean score (CI)	77.4 (76.4–78.3)

EQ-VAS, EuroQol visual analogue scale; CI, confidence interval.

**Table 4:** Key complications related to hybrid THR.

Variable, no.%	Hybrid THR (*n* = 124)
Revision (acetabular/stem/both)	2 (1.6)/6 (4.8)/1 (0.8)
Aseptic loosening (stem loosening)	10 (8.0)
Dislocation	6 (4.8)
Periprosthetic fracture	3 (2.4)

THR, total hip replacement.

## Discussion

Our study has several limitations that warrant careful consideration. First, its retrospective design inherently introduces biases, including potential selection and recall biases, evolving symptom and disease definitions over the extended follow-up period, and incomplete pre-fracture functional data. These factors, compounded by fragmented historical records, may limit the depth of analysis and introduce inaccuracies. Second, the multi-center nature of this study introduces variability in surgical practices—such as differences in surgical approaches, acetabular cup positioning, reaming techniques, and cement application protocols—which could confound outcome interpretations. Third, technological advancements during the 18-year study period (2005–2023) may have introduced temporal biases, as evolving implant designs and cementing methods could influence long-term survivorship metrics. Fourth, the observed rise in orthopedic complications beyond 5 years may reflect stricter diagnostic criteria rather than true incidence increases, though factors like bone cement degradation or weight-related stress cannot be discounted. Fifth, competing risks, such as mortality, may inflate Kaplan–Meier survival estimates, necessitating cautious interpretation. Finally, the observational framework precludes causal inferences. Despite these limitations, our findings provide critical insights into hybrid THR outcomes, underscoring the need for prospective, standardized studies to address these constraints and refine surgical protocols.

In patients aged 50–70 years undergoing hybrid THR following failed PFNA procedures, our study demonstrated robust long-term efficacy, with a 10-year revision-free survival rate of 87.1%, reinforcing hybrid THR as a reliable revision strategy for this demographic. Notably, stratified survival analysis revealed a significant age-dependent divergence: patients aged 50–60 years exhibited markedly superior outcomes compared to the 60–70-year cohort (*p* = 0.00026), highlighting the critical role of age stratification in preoperative risk assessment and surgical planning. As illustrated in Figure 6, non-infected individuals aged 50–60 achieved the highest 10-year survival rate (89.6%), whereas Staphylococcus-infected peers in the same age group showed a steep decline (72.3%). These findings not only validate the clinical utility of hybrid THR in mitigating complications such as stem loosening but also emphasize the necessity of tailored approaches for older patients with higher comorbidity burdens. By integrating these insights, clinicians can optimize patient selection and refine perioperative protocols to enhance longevity and functional outcomes in revision arthroplasty.

The observed trend towards improved survival rates over the decade is likely attributable to advancements in bone cement technology and the refinement of indications for cemented arthroplasty (23, 24). These advances have culminated in a reduced frequency of hip-related complications and enhanced patient-reported postoperative health outcomes (23, 25). Consistent with recent studies (5, 26, 27), our investigation found no significant deviations in 5-year survival rates, likely due to comparable follow-up durations across investigations. However, at the 10-year benchmark, we observed a marginal reduction in survival rates compared to previous data (16, 28). This variance may reflect elevated CCI scores, infection-related risks (e.g., Staphylococcus and Enterococcus infections), and a higher incidence of bone and soft-tissue complications arising from unsuccessful PFNA procedures within our cohort. The heightened CCI scores suggest an increase in competing risks, including mortality. Furthermore, the suboptimal survival rates associated with hybrid THR could be linked to disparities in prosthetic design and advancements in bone cement technology (15, 29, 30). While our study extends the follow-up period, it only partially corroborates the efficacies of hybrid THR, highlighting the absence of widely accepted guidelines for mitigating mechanical failures when converting from failed PFNAs to THRs. Variations in implant designs and material properties contribute to significant discrepancies in research outcomes, further perpetuating the debate over the long-term effectiveness of hybrid THR (31, 32).

Aseptic loosening remains a notable complication in revision surgeries involving PFNA (5, 14). Identifying specific causative factors for this complication in cement-fixed femoral components proves challenging (28, 33). Contributing elements include the enlargement of the medullary cavity, metal fatigue, inflammatory responses at the cement-bone interface, and alterations in stress distribution within the proximal femoral cortex (5, 34). Notably, infection status may exacerbate these mechanical stresses, particularly in older cohorts with compromised bone quality. Comparative studies have elucidated distinct mechanisms underlying aseptic loosening in acetabular and femoral components (35, 36). For the acetabular component, loosening is primarily attributed to biological responses (37), such as wear debris-induced macrophage activation and cytokine-mediated osteolysis (37, 28). In contrast, loosening of cement-fixed femoral components is largely linked to mechanical factors, including uneven distribution of bone cement and high cement porosity (38, 39). Hybrid THR, which has gained preference in contemporary arthroplasty, adopts a dual approach to address these issues (40, 41). It employs bone cement for immobilizing femoral components, effectively mitigating postoperative thigh pain and counteracting early sinking and loosening. Simultaneously, hybrid THR utilizes uncemented prostheses for the acetabulum, aiming to reduce the incidence of postoperative loosening. This bifurcated approach reflects an evolving strategy to optimize patient outcomes by addressing the distinct pathophysiological processes underlying aseptic loosening in different components of hip prostheses (9, 13, 33, 41).

The long-term effectiveness of THR prostheses varies, with not all patients deriving equal benefit from THR revisions (6, 9). To effectively address the rising incidence of primary orthopedic complications, it is imperative to consider both the biological characteristics of patients and the specific clinical indications for hybrid THR (8, 15). Despite recent calls for comprehensive research, initiating a nationwide survey to precisely define criteria for hybrid THR poses considerable challenges. The observed increase in the application of THR procedures remains somewhat enigmatic (13). This uptick may reflect either an expansion in clinical indications for THR or simply reflect individual clinician preferences (8, 6). However, the criteria currently guiding THR indications continue to rely heavily on individual clinician experience rather than a standardized, evidence-based approach (6, 8, 42, 43). This reliance complicates efforts to accurately assess the factors contributing to the observed increase in THR utilization. Thus, a more nuanced understanding of these dynamics is essential to clarify the roles of clinician judgment and evolving clinical guidelines in the rising adoption of THR procedures.

## Conclusion

Hybrid THR demonstrates substantial clinical efficacy as a revision strategy for patients aged 50–70 years with prior failed PFNA procedures, offering enhanced prosthesis longevity and reduced implant-related complications. This study highlights its capacity to improve postoperative health outcomes and mitigate risks such as stem loosening and periprosthetic fractures. Survival analysis stratified by age and infection status revealed a significant age-dependent disparity, with younger patients (50–60 years) achieving superior revision-free outcomes compared to older counterparts, while bacterial infections (Staphylococcus and Enterococcus) further exacerbated survival declines in vulnerable subgroups. These findings provide pivotal insights for refining surgical decision-making in revision arthroplasty. Future research should prioritize optimizing age-specific protocols and addressing long-term cement degradation to improve outcomes in aging populations with comorbidities.

## Data Availability

The original contributions presented in the study are included in the article/Supplementary Material, further inquiries can be directed to the corresponding author/s.
